# Alveolar Abscess

**Published:** 1867-01

**Authors:** J. L. Nourse


### DENTAL REGISTER.

VOL. XXI.] JANUARY, 1867. [No. 1.

### Original Communications.

### ALVEOLAR ABSCESS.

BY DR. J. L. NOURSE.

Read before the Central States Dental Association, July, 1866.

ABSCESS is a term derived from the Latin. "Ab," from, or away, and "Cedo," to go, to separate, or give up, and literally means the separation or yielding up of a part or principle that is to go out or be expelled, and in a general sense is applied to those pathological productions within the body, which are characterized by a separation and disorganization of a portion of the tissues, and the production and expulsion of offensive, purulent substance; but in a more limited and proper sense it is used to designate that particular form of the disease, which, usually situated within the fascia of some organ, is mainly dependent upon persistent local irritation, and sthenic or circumscribed inflammation for its production and continuance; and to distinguish it alike from simple phlegmon, or common boils, pimples, &c., which are naturally developed in the soft tissues, and are indiopathic in their character on the one hand; and, on the other, from ulcers.

By Alveolar Abscess, then, we are to understand a condition of suppuration within or upon the alveolar process, and having for its seat the periosteum of the alveolus, or the peridental membrane of a tooth. In order to fully comprehend the nature and character of an abscess, it will be necessary to examine, somewhat minutely, not only the immediate and remote causes upon which it depends, but also the physical characteristics of the parts most intimately concerned in its production, and some of the more important changes which take place during the progress of its formation.

Suppuration, as usually met with in alveolar, and all similar abscesses, in healthy subjects, is immediately dependant upon inflammation, or rather is one of the progressive stages of traumatic inflammation, in which important changes are effected in the blood and blood vessels, consequent upon a depressed state of vitality produced by local irritation. Whatever tends to produce this depression of vitality within the alveolar periosteum, whether by mechanical or chemical means, may be regarded as remote causes of alveolar abscess. Among these may be enumerated, contusion, such as might be produced by undue pressure upon a tooth; the presence of necrosed roots of teeth or other obnoxious foreign bodies; the introduction of acids, or other corrosive agents that destroy the tissues, or the infiltration of poisonous gases, such as are engendered by the putrefaction of a devitalized pulp. This last is, by far the most common.

As blood is the substance most intimately concerned in the formation of abscess and from which the materials are chiefly derived, and as the capillaries are the medium through which the necessary changes are effected, I propose, in the first place, to notice, as briefly as possible, some of the characteristics of these vessels, and also of those portions of the blood most actively engaged in the process, and to trace the most important changes that occur from the departure of the parts from a normal condition to the full development of an active abscess.

The capillaries, though but the continuation of the minute extremities of the arteries, from which they arise by a gradual and almost imperceptible transition, and as gradually merge again into the venous system, are justly entitled to the rank of a distinct system in view of the important differences which exist between their structure and functions, and those of the radical branches.

By their intricate ramifications, and repeated and innumerable anastomoses, they form an exceedingly delicate, continuous and uniform network which pervades every part of the organic structure; and thus enables these vessels to perform one of their important offices—that of conveying within reach of every particle of animal tissue, its necessary amount of nourishment, and also the materials for reproducing any portion that may have been accidentally destroyed.

As it is through the capillaries alone that the great process of nutrition is effected, they are provided with walls peculiarly adapted to the discharge of this important function. While the walls of the arteries are thick, strong, and perfectly impervious, those of the capillaries are extremely thin and elastic, and are endowed with a peculiar secreting power, by which the pabulum necessary for the nourishment of the part is taken up and prepared from the blood, and transmitted through them, to be assimilated into the tissue without. They are also endowed with a high degree of sensibility, by means of a liberal supply of delicate nerve branches, which enables them to respond promptly to the influence of the least stimulation, and, like the arteries, possess a strong contractile power, which materially affects the movements of the blood, and an impairment of which gives rise to those morbid changes which constitute that pathological condition known as inflammation.

Healthy blood consists of a thin, nearly colorless fluid, called *Liquor Sanguinis*; in which float freely a large number of red globules or corpuscles, together with a comparatively small number of white corpuscles.

The liquor sanguinis, or serum, is composed chiefly of water, albumen, fibrin, various salts, especially those of sodium and potassium, with fatty, extractive, and other matters. Of these, albumen and fibrin will claim our special attention.

Albumen is a whitish, nearly transparent substance, which, though it does not spontaneously coagulate, forms, in combination with certain agents, an insoluble compound, a property which we are enabled to turn to valuable account in the treatment of abscess. It is the raw material, so to speak, out of which much of the fibrin, and many other substances are elaborated. It exists in the blood, in a state of perfect solution, and is carried to all parts of the system by the circulation, and is taken up and appropriated wherever it may be required in the process of nutrition.

Fibrin, in healthy blood, moving in healthy vessels, is, like the albumen, in a state of perfect solution, and though it differs but slightly from the latter substance in its chemical composition, it is a step further advanced in the scale of vitality, and is far more perfectly prepared for conversion into organized tissue, and supplies, directly, a large portion of whatever is required for replenishing the natural waste, and for reproducing lost portions of nearly all the solid parts of the body.

It possesses the remarkable property of spontaneously coagulating, or passing from a fluid to a solid state, thus forming a fibrous structure which, under favorable circumstances, becomes the basis of living, organized tissue. It is exceedingly sensitive to the least excitement that may occur in the capillaries through which it may be passing; and any irritation in the vicinity of these vessels, whether chemical or mechanical, sufficient to endanger the vitality of a part, is the signal for the manifestation of its peculiar powers.

The presence of any irritating, or injurious principle being communicated to the walls of the capillaries by the nervous filaments which accompany them, a specific action is produced within their substance, which rapidly extends to the arteries. The contractile power of these vessels is at first stimulated into increased activity, by which the force of the circulation is considerably increased, and a more rapid flow of blood to the affected part produced.

This increase of the circulation, or determination of the blood, marks the first stage in the progress of inflammation.

The fibrin of the blood coming in contact with the excited walls of the capillaries, is arrested in its progress, seizes upon and assimilates to itself a portion of the albumen, by which means its quantity is largely increased, an irregular relaxation soon takes place in the blood vessels, portions of which swell out into pouches, admitting a larger amount of blood, and, at the same time, retarding its passage through them, the blood corpuscles accumulate and pack upon each other within the contracted portions of the capillaries, still further impeding the circulation, and finally, if the irritation is continued with sufficient violence, producing complete stagnation, or stasis.

This engorgement, or congestion, is the second stage in the progress of inflammation toward the formation of an abscess.

In the mean time the walls of the capillaries appear to have taken on exalted functional activity, and have elaborated from the liquor sanguinis a peculiar fibrinous fluid, called plastic, or coagulable lymph, which exudes, or is poured into the æreola, or other surrounding tissue, with a view to repair any injury the parts may have sustained, thus distending these tissues more or less, according to the violence of the irritation, and the density of the part affected, and producing the œdema or swelling which usually accompanies inflammation.

The periosteum of the teeth being confined within narrow limits, by the bony walls of the alveolus on one side, and the tooth on the other, admits of but little distension, and the pressure thus produced slightly elevates the tooth in its socket, and occasions severe, heavy, deepseated pain. This condition of the parts is known as *acute periostitis.*

The lymph thus exuded is a thin, transparent, and extremely viscid substance, capable of being drawn out into long, thin filaments, and consist of a thin serum, which contains a great number of globules, commonly called exudation corpuscles, and pervaded by exceedingly minute granules. The serum, like the liquor sanguinis, contains water, albumen, fibrin, &c. This lymph is not merely a transfusion of the serum of the blood consequent upon an excessive distension of the blood vessels, nor an effort of nature to simply relieve them of the unnatural pressure upon their walls, but is essentially a *secretion*, elaborated from the blood by a definite process of development, by which the fibrin itself has undergone a higher elevation, is still further advanced in the scale of vitality, and is now fully prepared to perform its important office of repairing an old or injured tissue, or producing a new one.

The first efforts of the lymph, after its infiltration into the inflamed periosteum of a tooth, is to so fortify and strengthen this tissue as to enable it to withstand the influence of the irritating agent, and to build up a barrier by which to circumscribe its destructive action. This it does by depositing within its substance an additional amount of newly organized material, thus increasing the density of the structure, and its power of resistance.

But the irritation continuing with sufficient violence to overcome and completely depress the vitality of the tissue, that particle most immediately within the influence of the destructive agent, and over which the vital forces have least power, gradually dies and disintegrates, and, at the same time, gives rise to a similar change in the effused lymph—a process by which fibrin looses its coagulating power, and is converted from a plastic, or organizable substance, to an aplastic or unorganizable one.

This change, thus effected, constitutes suppuration, and is the fourth stage of inflammatory action.

By this process a new substance is generated called pus, which is a thick, creamy, yellowish white, opaque, neutral fluid, free from smell, and is said to have a "sweetish mawkish taste, and consists of a thin serum called *liquor puris*, in which float a vast number of pus corpuscles. Its specific gravity is from 1,02 to 1,04, and analysis shows it to contain water, fat, albumen, and extractive matter, with a small per cent. of salts. The pus corpuscles, when examined under the microscope, are found to be smooth, opaque, spherical globules, measuring from 1-5000 to 1-2000 of an inch in diameter, or even larger, and are nearly indentical with the white corpuscles of the blood, and the lymph corpuscles of the chyle, from which they can be distinguished only by the two, three or four central nuclei, which may be seen when they are rendered transparent by the action of acetic acid.

The matter thus produced forms a center, or nucleus, from which the disorganizing process extends to the adjoining particles, which, in turn, are broken down and transformed into pus. In this way a portion of the periosteum of the tooth is destroyed, denuding that part of the root, thereby depriving it of this, probably its only remaining source of nutrition, and rendering it still more obnoxious to the system. Meantime, the surrounding tissue which is less inflamed, and in which the vitality is impaired but not destroyed, becomes so consolidated, by the deposition of newly organized matter, as to form a wall or sack, and thus prevent the infiltration of the pus into its substance, and so, in a measure, retard the progress of this destructive process.

This sack, when its formation is completed, with its contents, is an ALVEOLAR ABSCESS.

It continues to gradually advance however, in that direction which offers the least resistance, or seems most favorable to its extension, until an opening is formed into some natural cavity, or upon some free surface, through which the accumulated matter may be discharged. The root of the tooth being dense in structure, and not easily broken down, its progress in this direction is slow; the periosteum being strongly fortified, is enabled to withstand, to some extent, its encroachments, and generally rallies sufficiently to prevent its extension far into its substance; the alveolus, being soft and porous, more readily succumbs to its attacks, and affords a more ready transit, and consequently, instead of advancing through the body of the tooth, or following the periosteum to the margin of the gum, its course is most commonly through the process, to the nearest external surface, thus forming a fistulous channel through which the discharge may be kept up indefinitely, or until the irritating cause is removed, or so far allayed that the vital forces are enabled to overcome and control it sufficiently to permit the parts to perform their normal functions.

The eruption of the abscess is usually followed by a partial subsidence of the inflammation, the capillaries recover their contractile power, and the circulation of the blood is resumed. Within the body of the abscess, however, the morbid condition still exists, and although its dimensions are not afterwards materially increased, the albumen which still exudes from the capillaries, and which the fibrin is unable to appropriate, is rapidly assimilated into pus, which again accumulates, and is again discharged, thus constituting a *chronic abscess.*

The nature and *modus operandi* of the formation of an abscess being clearly understood, the condition necessary to be secured to effect a cure may be readily recognized. These, together with the means by which they may be obtained, may be comprehended in a few general principles.

The first is the removal of the existing cause. If this is a tooth whose fang has been so far deprived of its investing membrane, and consequently of its vitality, as to render its presence obnoxious to the living parts, there should be no hesitation in deciding upon its immediate extraction.

If, however, the irritation is dependant upon changes which are yet taking place within the pulp cavity, and it is desirable to preserve the tooth, a free opening should be made into the cavity, by which access may be had to all its parts, and every particle of dead or decomposing matter removed from its entire length, and the foramen at the apex of the root enlarged sufficiently to afford access to the interior of the sack.

The second condition to be secured is a healthy action within the walls of the sack and the fistulous channel of the abscess, or, according to Hunter, "adhesive inflammation."

This is effected by relieving the sack of its purulent contents, and introducing some therapeutic agent that, by uniting with the superabundant albumen, will arrest the suppurative process, and promote the organization of the lymph.

Among the articles used for this purpose may be mentioned—Tanic Acid, Nitrate of Silver, Iodine, and Creosote.

Tanic acid forms a very permanent compound with albumen, but its action is too slow; and the difficulty of introducing it in a concentrated form too great to meet the requirements of many cases.

Nitrate of silver unites with albumen, but the compound is not so permanent, and the powerful caustic properties of the nitric acid, liberated in the process, somewhat impairs its usefulness.

Iodine is very useful in many cases; but its escharotic properties, and the rapidity with which it is absorbed, are somewhat objectionable; combined with creosote it is a valuable agent.

Creosote, in addition to its strong affinity for albumen, with which it rapidly forms a permanent, insoluble compound, possesses the valuable property of arresting and preventing the decomposition of animal matter, which renders it preferable to any other agent that has hitherto been introduced for the treatment of abscess. Its great penetrating power enables it to pervade every part of the cavity, and to diffuse itself over the entire surface of the sack, thus effectually securing the desired result. It is also an active stimulant, which still further enhances its value. It may be readily introduced by injecting into the fistulous opening, or through the pulp cavity of the tooth—the latter is usually the more convenient and better way—this should be repeated from time to time, until the symptoms indicate the healty action desired.

The third condition to be brought about, and which naturally follows when the preceding one is established, is the restoration of the part destroyed, and the absorption of the sack.

This is readily effected by the recuperative powers of nature, aided by such stimulation as is afforded by the agent injected.

The albumen being coagulated upon the internal surface of the sack, is prevented from further conversion into pus, and forms a protecting coat, beneath which the fibrin spontaneously coagulates into fibrous loops, in which the granules arrange themselves, by their own vital impulses, into groups of neuclei, which are rapidly converted into cells. Into this structure blood vessels and nerve filaments are projected from the neighboring branches, thus completing its organization. This process is continued until the cavity occupied by the abscess, external to the root of the tooth, is filled with living, healthy tissue.

The fourth and last condition to be secured is one in which all liability to a recurrence of the disease will be, as far as possible, prevented, and simply consists in so thoroughly filling the canal and pulp cavity of the tooth with some suitable material as to perfectly exclude all exciting or irritating agents.

These conditions, successively obtained, will afford a good foundation on which to base a hope of a permanent cure.

Although, as before stated, abscesses are mainly dependent upon local causes for their production, and may appear whenever the conditions necessary for their formation are fulfilled, there are certain conditions of the system, in general, which seem peculiarly favorable to their development, and which materially retard the progress of their cure, and increase the liability of their recurrence.

When an abscess occurs in a healthy subject, the depression of vitality and abnormal condition of the blood are confined to the point of local irritation. The prognosis in this case is highly favorable, and the local treatment just indicated will generally prove successful in subduing it and restoring the parts to perfect health. But we frequently meet with cases in which the blood is in a low, morbid, deteriorated condition, consequent upon impaired or vitiated nutrition, imperfect assimilation, inhalation of impure air, epidemic influences, or upon some peculiar, depressing diathesis.

In this condition of the blood, there is a decidedly increased tendency to purulent transformation, which is likely to be developed by very slight local irritation, and its activity will vary according to the nature and degree of the departure of the blood from its normal condition. If this departure is occasioned only by a temporary derangement of the nutritive process, or by miasmatic, or epidemic influences, an effort is usually made to purify itself by means of boils, and a slight morbid excitement in the periosteum which would otherwise produce but a mild form of periostitis, now degenerates into an active abscess, and hence, not only the frequency of its occurrence, but the difficulty of subduing it, and the liability of its recurrence are greatly increased, and in addition to the usual local treatment, the employment of such therapeutic agents and such regimen as is best calculated to restore the blood to a healthy condition, is clearly indicated.

But when this deterioration of the blood is occasioned by some depressing diathesis, as the scrofulous or syphilitic, the purulent formation sometimes approaches an ulcerative, or gangrenous character, according to the nature and activity of the constitutional disease. When alveolar abscess occurs in a subject thus tainted, the prognosis is extremely unfavorable, and, in the language of Prof. Harris, "the only treatment that can be successful is preventive rather than curative, for the latter, to be successful, calls for the loss of the offending tooth. Another condition of the system, worthy of consideration in this connection, is that very common, but somewhat obscure one, frequently produced by sudden thermal and other changes in the atmosphere (and sometimes most unaccountable), by that mysterious process so appropriately expressed by that unmeaning phrase, "Catching Cold."

Very little is really known of the precise nature of the pathological changes that take place, or the manner in which they are effected; but it is quite evident that in consequence of the disturbance in the animal heat, there is a depression of the vital forces and a relaxed condition of the system—states highly favorable to the production of inflammation and purulent transformation. It is also quite certain that it exerts a very unfavorable influence in the treatment of abscess. The symptoms are more or less aggravated, and the best efforts to so effect a cure are often baffled; and while we are yet congratulating ourselves upon the completion of a successful operation, an unfavorable change in the weather, or a sudden bad cold, will sometimes arouse into renewed activity the subdued inflammation, and snatch from us the triumphs of our fancied victory.

These facts would seem to indicate that the state of the weather should not be wholly disregarded, and that we may sometimes profitably postpone the completion of an operation until the barometer indicates a favorable condition of the atmosphere, and it would be well also, to caution a patient against any unnecessary exposure, and to avoid, as much as possible, all liability to "take cold" until the parts have had time to recover health and strength sufficient to withstand its depressing influence.

In conclusion, I will only add that the great and constantly increasing prevalence of this destructive disease, calls for renewed diligence in the study of its nature and treatment, and the free and liberal diffusion of all the light afforded by science to illuminate our pathway to the achievement of more completeness in its management.

